# Sarcopenia as a Prognostic Factor for Critical Limb Ischemia: A Prospective Cohort Study

**DOI:** 10.3390/jcm14155388

**Published:** 2025-07-31

**Authors:** Paula Luque-Linero, Emilio-Javier Frutos-Reoyo, Luis Castilla-Guerra, Miguel-Ángel Rico-Corral, Prado Salamanca-Bautista, Fernando Garrachón-Vallo

**Affiliations:** 1 Department of Internal Medicine, Hospital Universitario Virgen Macarena, 41009 Seville, Spain; paula.luque.linero.sspa@juntadeandalucia.es (P.L.-L.); castillafernandez@hotmail.com (L.C.-G.); marc@us.es (M.-Á.R.-C.); msalamanca2@us.es (P.S.-B.); fgarrachon@gmail.com (F.G.-V.); 2 Department of Rehabilitation and Physical Medicine, Hospital Universitario del Río Hortega, 47012 Valladolid, Spain; 3 Department of Medicine, Universidad de Sevilla, 41004 Sevilla, Spain

**Keywords:** sarcopenia, chronic limb-threatening ischemia, mortality, amputation, calf circumference, frailty, prognosis

## Abstract

**Introduction and Aim:** Sarcopenia has emerged as a key prognostic factor in patients with chronic limb-threatening ischemia (CLTI), with potential implications for clinical decision-making. This study aimed to assess the association between sarcopenia and clinical outcomes, mortality, and amputation, using simple, accessible screening tools in a CLTI population. **Methods:** In this prospective, single-center study conducted between December 2023 and December 2024, 170 patients with CTLI were enrolled. Sarcopenia screening was performed using the SARC-F (strength, assistance in walking, rising from a chair, climbing stairs, falls) questionnaires, handgrip strength measurement, and calf circumference, adjusted for body mass index and sex. The primary outcome was 6-month all-cause mortality and/or major amputation. **Results:** Sarcopenia was identified in 77 patients (45.3%). Compared to non-sarcopenic individuals, sarcopenic patients were significantly older. They exhibited greater functional impairment, as well as poorer nutritional and muscle status. They also had significantly higher in-hospital mortality (16.9% vs. 3.2%, *p* = 0.002), 30-day mortality (24.7% vs. 4.3%, *p* = 0.001), and 6-month mortality (50.6% vs. 15.1%, *p* = 0.001). Sarcopenia was significantly associated with the primary outcome in univariate analysis (HR: 2.05; 95% CI: 1.31–3.20; *p* = 0.002) and remained an independent predictor after multivariate adjustment (HR: 1.95; 95% CI: 1.01–3.79; *p* = 0.048). **Conclusions:** Sarcopenia is a strong, independent predictor of poor outcome in patients with CLTI. Its detection through simple tools offers an easy and cost-effective strategy to improve risk stratification and guide early intervention through exercise-based therapy.

## 1. Introduction

Critical limb-threatening ischemia (CLTI) represents the terminal stage of peripheral arterial disease (PAD) [1]. It is defined by the presence of rest pain and/or non-healing ulcers or infections in the affected limbs [1]. This condition carries a poor prognosis, characterized by a high risk of limb amputation, an increased likelihood of major adverse limb events (MALEs), major adverse cardiovascular events (MACEs), and all-cause mortality [2].

The Society for Vascular Surgery recognized the importance of enhancing risk stratification for amputations and evaluating treatment outcomes. To address this, they developed the WIfI classification, which focuses on three key factors: wound, ischemia, and foot infection [3]. In 2020, a complementary tool was introduced, the Global Limb Anatomic Staging System (GLASS), to predict the technical success and long-term patency of arterial revascularization based on the extent and distribution of atherosclerotic lesions [4]

However, the severity of CLTI extends beyond the risk of amputation. Patients with this condition are significantly more likely to suffer major cardiovascular events and mortality within a year. Unfortunately, existing tools like the WIfI classification, the GLASS, and traditional cardiovascular risk factors fully explain the variations in patient outcomes. In recent years, sarcopenia has emerged as a promising prognostic factor in patients with advanced PAD, potentially offering new insights into risk prediction and management [5].

Sarcopenia is characterized by progressive loss of skeletal muscle volume and progressive reduction in skeletal muscle function [6] and is associated with frailty, increasing age, poor psychological reserve, and chronic illness. Since 2019, growing interest in sarcopenia has arisen from observations that patients with reduced muscle strength and mass experience worse clinical outcomes [7]. The negative correlation between sarcopenia and overall survival has been discussed and shown in other diseases, including malignancies, post-liver transplantation, abdominal aortic aneurysms, and, without exception, peripheral arterial disease [5].

Both sarcopenia and CLTI are clinical entities independently associated with extremely poor prognosis, including high rates of mortality, cardiovascular events, and limb loss [8,9]. Despite this, both conditions remain significantly underdiagnosed and undertreated in everyday practice.

In recent years, several studies have investigated the relationship between sarcopenia and adverse outcomes in patients with CLTI. Nearly all of these studies have demonstrated a direct association between sarcopenia, CLTI, and poor prognosis, defined as mortality, major vascular events, or limb amputation [9,10,11,12,13,14].

In this context, the management of sarcopenia in patients with CLTI should be multimodal and involve a multidisciplinary team [5]. Therefore, besides revascularization, the best therapeutic strategy must include exercise [11]. This approach should include structured exercise programs, among other interventions. However, the essential first step toward effective treatment is a timely and accurate diagnosis.

To assess muscle mass, the majority of studies have relied on complex and costly imaging-based techniques such as computed tomography (CT), magnetic resonance imaging (MRI), dual-energy X-ray absorptiometry (DXA), or bioelectrical impedance analysis (BIA) [6]. While accurate, these methods are not always feasible in routine clinical practice due to limited accessibility and higher resource requirements [5,12,13,15]. In response to these limitations, expert consensus statements have sought to clarify and standardize alternative approaches to diagnosing sarcopenia, emphasizing the importance of combining measures of muscle strength and function with more accessible proxies of muscle mass.

Among these, calf circumference has emerged as a practical, low-cost, and reproducible surrogate marker that correlates reasonably well with total muscle mass, particularly in older adults and hospitalized patients [16,17]. Recent clinical guidelines have proposed a simpler and more practical alternative for identifying sarcopenia: the measurement of calf circumference adjusted for body mass index (BMI), which has shown promise as a reproducible and accessible surrogate [18].

Our study aims to evaluate the relationship between sarcopenia and adverse outcomes, defined as amputation or death in patients with CTL, using a simple, low-cost, and reproducible method of sarcopenia assessment based on functional and anthropometric parameters.

## 2. Material and Methods

### 2.1. Study Design and Population

This was a prospective, observational study conducted over 12 months at Virgen Macarena University Hospital in Seville, Spain, from December 2023 to December 2024. This study enrolled patients admitted to Internal Medicine wards with a confirmed diagnosis of critical CLTI, according to the ESC Guidelines for Peripheral Arterial Disease published in 2024 [1].

### 2.2. Participants

Eligible participants were adults (≥18 years) diagnosed with CLTI of atherosclerotic origin and had signed informed consent. Exclusion criteria included acute limb ischemia, non-atherosclerotic etiologies, active malignancy, autoimmune or systemic inflammatory diseases, and inability to participate in clinical evaluations. Patients were consecutively recruited during hospital admissions.

### 2.3. Data Collection and Clinical Assessment

Baseline data were collected, including demographics, vascular risk factors (e.g., diabetes mellitus, hypertension, dyslipidemia, smoking status), comorbidities, and current medications. The severity of CLTI was classified using the Fontaine staging system. Additionally, all participants were assessed with standardized scales to evaluate frailty, nutritional status, and the degree of dependency. Specifically, the Barthel Index was used to assess dependency, the Mini Nutritional Assessment–Short Form (MNA-SF) was used to evaluate the risk of malnutrition, and the SARC-F (strength, assistance in walking, rising from a chair, climbing stairs, falls) questionnaire was used to screen for sarcopenia. Follow-up was performed to record the occurrence of major clinical outcomes—major limb amputation and all-cause mortality. Major amputation was defined as any surgical limb removal performed proximally to the ankle joint, including below-knee (transtibial) and above-knee (transfemoral) amputations. Minor amputations, such as toe or forefoot resections, were not included in this definition.

### 2.4. Sarcopenia Evaluation

The assessment of sarcopenia followed the criteria of the European Working Group on Sarcopenia in Older People (EWGSOP) [5]. A three-step approach was applied, as follows:

1. Initial Screening: The SARC-F questionnaire, a validated tool for identifying the risk of sarcopenia, was administered to all participants. This tool includes five components (strength, assistance in walking, rising from a chair, climbing stairs, and falls), scored from 0 to 10. A total score ≥4 was considered indicative of sarcopenia risk.
2. Muscle Strength Assessment: Patients with a positive SARC-F underwent grip strength testing using a Holtein digital handgrip dynamometer (KEEDA brand), following the Southampton protocol [9]. Participants were seated, with feet flat on the floor, elbow flexed at 90 degrees, wrist in a neutral position, and shoulder adducted. They were instructed to squeeze the device with maximum effort for 3 s. The test was repeated three times on each hand, and the highest value was recorded. Strength values were compared to age- and sex-specific percentile charts validated for the local population. A grip strength below the 10th percentile (P10) was considered indicative of probable sarcopenia.
3. Confirmation of Sarcopenia: For participants with reduced muscle strength, muscle mass was estimated by measuring calf circumference (CC), adjusted for the body mass index (BMI). This method is a simple and practical alternative to imaging. CC was measured at the widest point of the non-dominant calf using a non-elastic, flexible measuring tape, with the patient in a seated and relaxed position. A low CC adjusted for BMI was considered indicative of reduced muscle mass, confirming the diagnosis of sarcopenia.

For the diagnosis of sarcopenia, three criteria were required. First, the SARC-F questionnaire was administered, with a score ≥ 4 considered positive. Second, handgrip strength below the 10th percentile, stratified by sex and age, was classified as reduced muscle strength. Third, calf circumference adjusted for the BMI was measured, with values below the established threshold considered indicative of low muscle mass. Only participants who met all three criteria were classified as sarcopenic.

### 2.5. Statistical Analysis

Continuous variables were expressed as mean ± standard deviation (SD) or median and interquartile range (IQR), depending on whether they followed a normal distribution. Categorical variables were presented as the number of patients and percentages. Normality of the data distribution was assessed using the Kolmogorov–Smirnov test. Comparisons between sarcopenic and non-sarcopenic patients were made using Student’s *t*-test or Mann–Whitney U test for continuous variables and Chi-square or Fisher’s exact test for categorical variables. Kaplan–Meier curves and the log-rank test were used to compare event-free survival between groups, particularly assessing the impact of sarcopenia. Associations between 6-month all-cause mortality and/or major amputations were estimated in a multivariate Cox regression model that included variables based on clinical relevance and/or statistical significance in the univariate analysis. The results were expressed as a hazard ratio (HR) with 95% confidence interval (CI). A *p*-value < 0.05 was considered statistically significant. Data analysis was performed using SPSS version 18.0 (IBM Corp., Armonk, NY, USA).

### 2.6. Ethical Aspects

This study was carried out in accordance with the Declaration of Helsinki. The study was approved by the Clinical Research Ethics of Sevilla (Internal Code 1293, approval date: 29 November 2023). Informed consent was obtained from all participating subjects.

## 3. Results

The study included 170 patients with CLTI, with a mean age of 72 years and a minority of women (25.9%). A total of 45.3% (n = 77) were identified as having sarcopenia. Sarcopenic patients were significantly older than non-sarcopenic patients (76 ± 12 vs. 68 ± 10 years, *p* = 0.001) and included a lower proportion of males (57.1% vs. 88.2%, *p* = 0.001).

No significant differences were observed between groups regarding rural residence. As for vascular risk factors, hypertension was more prevalent among sarcopenic patients (89.6% vs. 78.5%, *p* = 0.052), although the difference was not statistically significant. The prevalence of type 2 diabetes, dyslipidemia, and obesity was similar across groups.

Interestingly, active smoking was more common in the non-sarcopenic group (62.8% vs. 54.5%, *p* = 0.003), and alcohol use was significantly more prevalent in non-sarcopenic patients (34.4% vs. 18.2%, *p* = 0.018).

Among other medical comorbidities, atrial fibrillation was more prevalent in non-sarcopenic patients (18.3% vs. 35.1%, *p* = 0.013). A history of cancer was also significantly higher in the sarcopenic group (26% vs. 10.8%, *p* = 0.010). No significant differences were observed in rates of ischemic heart disease, stroke, chronic kidney disease, or heart failure.

Sarcopenic patients had significantly lower Barthel Index scores (median 40 vs. 90, *p* = 0.001), higher SARC-F scores (median 7 vs. 3, *p* = 0.001), and worse nutritional status per MNA-SF (median 5 vs. 9, *p* = 0.001).

In terms of muscle and anthropometric parameters, sarcopenic patients had markedly lower grip strength (9 kg vs. 23 kg, *p* = 0.001) and calf circumference (29 cm vs. 32 cm, *p* = 0.001).

There were no significant differences in the anatomical location of arterial disease or the Fontaine classification between groups. Infection rates were slightly higher in the sarcopenic group, but without statistical significance.

All these characteristics are shown in Table 1.

Clinical outcomes showed that sarcopenic patients had significantly worse prognoses. While total amputation rates were similar at admission (39% vs. 39.8%, *p* = 0.91) and 6 months (58.4% vs. 57%, *p* = 0.849), sarcopenic patients underwent fewer endovascular procedures (31.2% vs. 49.5%, *p* = 0.016) and less surgical debridement (14.3% vs. 29%, *p* = 0.022).

In-hospital mortality was significantly higher in the sarcopenic group (16.9% vs. 3.2%, *p* = 0.002), as was 30-day (24.7% vs. 3.2%, *p* = 0.002) and 6-month mortality (50.6% vs. 15.1%, *p* = 0.001). MACE rates were higher in sarcopenic patients during hospitalization (26% vs. 6.5%, *p* = 0.001) and at 6 months (67.7% vs. 21.5%, *p* = 0.001). MALEs also occurred more frequently in sarcopenic patients during hospitalization (77.4% vs. 55.8%, *p* = 0.003). Hospital stay duration did not significantly differ (median 9 vs. 11 days, *p* = 0.24). A significantly higher incidence of the composite endpoint of death or major amputation at 6 months was observed in patients with sarcopenia compared to those without. Specifically, 75.3% of patients with sarcopenia experienced this adverse outcome, compared to 43.0% of those without sarcopenia (*p* = 0.001). The management and outcomes of patients in the sarcopenic and non-sarcopenic patient groups have been detailed in Table 2.

In the univariate analysis, male sex (HR: 1.66; 95% CI: 1.04–2.66; *p* = 0.033), depression (HR: 2.11; 95% CI: 1.20–3.71; *p* = 0.009), and sarcopenia (HR: 2.05; 95% CI: 1.31–3.20; *p* = 0.002) were significantly associated with all-cause mortality and/or major amputation at 6 months. In the multivariate analysis, in addition to these variables, chronic kidney disease and male sex were also included, as these are factors related to mortality in other studies.

Using Cox proportional hazards regression, depression (HR: 3.23; 95% CI: 1.38–7.57; *p* = 0.007) and sarcopenia (HR: 1.95; 95% CI: 1.01–3.79; *p* = 0.048) remained independently associated with the composite outcome.

The results of the univariate and multivariate analysis are shown in Table 3.

**Figure 1:**
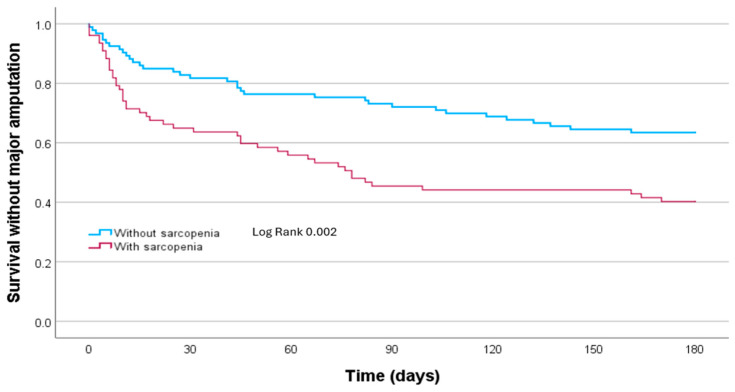
Kaplan–Meier curve for survival without major amputation at 6 months according to the presence of sarcopenia.

Survival analysis using Kaplan–Meier curves showed that patients with sarcopenia had higher mortality or need for major amputation at 6 months of follow-up compared to those without sarcopenia (Log-Rank *p* = 0.002) (Figure 1).

## 4. Discussion

Our study highlights sarcopenia as an independent prognostic factor for 6-month mortality and major amputation in patients with CLTI. This finding emphasizes the clinical importance of identifying and addressing the progressive loss of muscle mass and function in this high-risk population. Notably, the association between sarcopenia and poor outcomes remained significant even after adjusting for potential confounders such as age, chronic kidney disease (CKD), sex, and depression. These results are consistent with previous research reporting sarcopenia as an independent predictor of mortality and adverse limb events in patients with CLTI [19].

In the multivariate Cox regression model, we included variables that reached statistical significance in univariate analysis (*p* < 0.05), namely sex, sarcopenia, and depression. Additionally, age and CKD were incorporated due to their recognized prognostic value in CLTI [20], despite not showing formal significance in our univariate model. Among these, only sarcopenia and depression remained independently associated with adverse outcomes. This underscores their clinical relevance as potentially modifiable risk factors.

The role of depression in CLTI outcomes is increasingly recognized. Our findings align with those of Zielke et al. [21] and Harris et al. [22], who demonstrated that depression significantly increases the risk of major amputation and mortality, particularly in patients not receiving antidepressant therapy. Depression has also been associated with longer hospital stays and increased healthcare costs, and may negatively influence biological processes such as inflammation, tissue repair, and vascular health [23]. These insights suggest that depression is not merely a psychosocial comorbidity but a factor with direct physiological consequences in patients with vascular disease.

While male sex was associated with a worse prognosis in the unadjusted analysis, this association lost significance after adjustment. Interestingly, although the overall cohort had more male patients, sarcopenia was proportionally more prevalent among women. This contrasts with previous studies where sarcopenia was more commonly observed in men, likely due to cohort differences in sex distribution and muscle mass [12]. Still, as in other studies, we confirmed that increasing age was strongly associated with sarcopenia [13,24]. These demographic patterns suggest that the prognostic impact of sarcopenia may be influenced by age and sex and highlight the importance of considering patient-specific factors in clinical decision-making [25].

It is important to note that, unlike most previous studies, our work incorporated the EWGSOP2 [6] consensus criteria, using calf circumference adjusted for BMI as a practical and validated proxy for muscle mass assessment. In contrast, prior publications predominantly relied on computed tomography (CT)-derived measures of skeletal muscle area or density to define sarcopenia, without applying the SARC-F questionnaire or similar screening tools [5,12,15]. While the EWGSOP2 guidelines recommend SARC-F as an initial screening instrument to identify individuals at risk of sarcopenia, none of these studies reported using SARC-F or comparable functional questionnaires in their protocols. Therefore, our approach not only aligns with the updated European consensus definition but also demonstrates that simpler anthropometric assessments can effectively identify sarcopenia in clinical practice, especially in settings where advanced imaging modalities are not routinely available.

Another difference between our study and those that have been published is the study objectives. Previous studies have mainly focused on the association of sarcopenia with limb salvage [5], procedural outcomes [13], and overall mortality following revascularization [12]. In contrast, our primary objective was to evaluate sarcopenia as an independent prognostic factor for short- and medium-term all-cause mortality and amputation, irrespective of the revascularization strategy. This provides complementary evidence regarding the prognostic impact of sarcopenia beyond limb outcomes alone.

In terms of clinical outcomes, our results are consistent with several previous studies indicating that sarcopenia is associated with higher short-term and long-term mortality, amputation, and an increased risk of major adverse cardiovascular events (MACEs) [5,13]. For instance, Cao et al. demonstrated that sarcopenia independently predicted worse three-year survival following endovascular revascularization in patients with CLTI [5]. Similarly, a systematic review by Ferreira et al. confirmed lower overall survival and higher cardiovascular event rates among sarcopenic patients [19]. Notably, Taniguchi et al. [5] emphasize that sarcopenia should be regarded as a critical factor when deciding between conservative and aggressive interventions, particularly in high-risk populations [26].

In line with this perspective, our findings highlight the critical importance of incorporating structured, exercise-based therapies, particularly Supervised Exercise Therapy (SET), into the standard care of patients with CLTI and sarcopenia. The 2024 AHA/ACC guidelines on peripheral artery disease [1] give SET a Class I recommendation, recognizing its efficacy in improving walking capacity, muscle strength, and cardiovascular health, even in advanced stages of PAD [27]. Evidence from the SET Study [10,25,28] supports its role in enhancing physical performance, reducing inflammation, and improving endothelial function, mechanisms that may counteract both ischemic progression and sarcopenia. Although most studies focus on claudication, recent data suggest that properly adapted exercise programs can also benefit patients with CLTI, especially when multimodal approaches (resistance, balance, and functional training) are employed. SET also improves systemic metabolic parameters, such as glycemic control and blood pressure. Given that sarcopenia is now recognized as an independent predictor of mortality and cardiovascular events in CLTI, early identification and initiation of SET could play a pivotal role in improving outcomes. Importantly, exercise also provides significant mental health benefits. A recent BMJ network meta-analysis [29] found that walking, yoga, and strength training lead to moderate-to-large reductions in depressive symptoms, comparable to psychotherapy or antidepressants [30], and are effective even in those with comorbidities. Given that depression is a recognized independent predictor of mortality and cardiovascular events in CLTI, early identification and initiation of SET may yield dual benefits, physical and psychological. The multidisciplinary approach endorsed by current guidelines, combining vascular care, nutrition, rehabilitation, and internal medicine, provides the ideal framework for personalized exercise interventions. Rather than being viewed as adjunctive, exercise therapy should be considered a cornerstone treatment that addresses both ischemic burden and associated depression in this high-risk population [1,27].

Finally, it is important to highlight that one of the main strengths of our study lies in the use of simple, low-cost, and easily reproducible screening tools, such as adjusted calf circumference and handgrip strength, for the identification of probable sarcopenia in patients with CTLI. There are no other studies that have used calf circumference adjusted for BMI to assess sarcopenia in this patient population [31]. These basic assessments can be readily implemented in routine clinical settings and may allow for early recognition of patients at higher risk, thus facilitating timely intervention [18].

However, our study also has certain limitations. It was conducted in a single center, which may affect the generalizability of the results. Moreover, follow-up assessments of sarcopenia status or the implementation of targeted interventions, such as nutritional support or exercise programs, were not systematically performed during subsequent visits. Future studies should address these aspects to further elucidate the longitudinal impact of sarcopenia and the potential benefits of early therapeutic strategies.

## 5. Conclusions

Sarcopenia is a strong, independent predictor of mid-term mortality and major amputation in patients with CLTI. It can be easily assessed using simple tools such as adjusted calf circumference. Along with depression, it was more strongly associated with adverse outcomes than traditional risk factors. Given its easy assessment through tools like calf circumference, early identification and exercise-based interventions should be integrated into routine care.

## Figures and Tables

**Table 1:** General characteristics of patients with sarcopenia vs. without sarcopenia.

General Characteristics	Total Population (n = 170)	With Sarcopenia (n = 77)	Without Sarcopenia (n = 93)	*p*
Age (years)	72 ± 12	76 ± 12	68 ± 10	0.001
Male sex	126 (74.1)	44 (57.1)	82 (88.2)	0.001
Rural area	99 (58.2)	44 (57.1)	55 (59.1)	0.79
**Cardiovascular risk factors**				
Hypertension	142 (83.5)	69 (89.6)	73 (78.5)	0.05
Diabetes	129 (75.9)	59 (76.6)	70 (54.3)	0.84
Dyslipidemia	101 (59.4)	43 (55.8)	58 (62.4)	0.39
Active smoking	77 (45.3))	42 (54.5)	71 (62.8)	0.003
Obesity > 30	24 (14.1)	18 (23.4)	20 (52.6)	0.77
BMI (kg/m^2^)	25 (21–27)	25 (22–31)	26 (24–29)	0.80
**Medical history**				
Alcoholism	46 (27.1)	14 (18.2)	32 (34.4)	0.018
Ischemic heart disease	48 (28.2)	22 (28.6)	26 (28)	0.93
Stroke	42 (24.7)	21 (27.3)	21 (27.3)	0.48
Chronic Kidney disease	52 (30.6)	27 (35.1)	25 (26.9)	0.25
Heart failure	36 (21.5)	18 (23.4)	18 (19.4)	0.31
Atrial fibrillation	44 (25.9)	27 (35.1)	17 (18.3)	0.01
Autoimmune disease	10 (5.9)	5 (6.5)	5 (5.4)	0.76
COPD	19 (11.2)	9 (11.7)	12 (12.9)	0.85
Depression	22 (12,9)	11 (14.3)	11 (11.8)	0.63
Microvascular disease	43 (25.3)	16 (20.8)	16 (20.8)	0.22
History of cancer	30 (17.6)	20 (26)	10 (10.8)	0.01
Sleep apnea	13 (7.6)	3 (3.9)	10 (10.8)	0.09
**Scales**				
BARTHEL index	80 (40–96)	40(20–80)	90 (80–100)	0.001
SARC-F score	5 (3–8)	7 (5–9)	3 (1–5)	0.001
MNA-SF score	7 (4–10)	5 (3–7)	9 (7–11)	0.001
**Nutritional parameters**				
Handgrip strength (Kg)	23 (18–30)	9 (5–15)	25 (19–32)	0.001
Calf circumference adjusted for BMI (cm)	31.74 ± 5.93	29.43 ± 5.85	32.38 ± 0.48	0.001
**Location**				
Infrapopliteal lesions	61 (35.9)	28 (36.4)	33 (35.5)	0.78
Suprapopliteal lesions	21 (14.2)	8 (10.4)	13 (14)	0.77
Both locations	88 (51.8)	41 (53.2)	47 (50.5)	0.90
**Fontaine stage**				0.52
Fontaine stage III	73 (42.9)	31 (40.3)	42 (45.2)	
Fontaine stage IV	97 (57.1)	46 (59.7)	51 (54.8)	
**Complications**				
Infection	97 (57.1)	48 (62.3)	49 (52.7)	0.21

Quantitative variables are expressed as mean ± standard deviation or median (interquartile range), and categorical variables as number (percentage). BMI: body mass index; COPD: chronic obstructive pulmonary disease; MNA-SF: Mini Nutritional Assessment–Short Form; SARC-F: Strength, Assistance in walking, Rise from a chair, Climb stairs, Falls.

**Table 2:** Management and outcomes in patients with and without sarcopenia.

Event	Global Cohort	With Sarcopenia	Without Sarcopenia	*p*
Conservative treatment	41 (24.1)	23 (29.9)	19 (20.4)	0.11
Amputation	66 (39.4))	30 (39)	37 (39.8)	0.91
Minor	34 (20.0)	17 (22.1)	18 (51.4)	
Major	33 (19.4)	13 (40.6)	19 (59.4)	
Surgical debridement	38 (22.4)	11 (14.3)	27 (29)	0.02
Endovascular treatment	70 (41.2)	24 (31.2)	46 (49.5)	0.02
Ischemic heart disease	10 (5.9)	7 (9.1)	3 (3.2)	0.11
MACEs	27 (15.9)	20 (26)	6 (6.5)	0.001
MALEs	113 (63.5)	43 (55.8)	72 (77.4)	0.003
Death during admission	16 (9.4)	13 (16.9)	3 (3.2)	0.002
30-day mortality	26 (15.3)	19 (24.7)	18 (23.4)	0.002
Hospital stay (days)	10 (7–15)	9 (6–15)	11 (7–15)	0.24
Amputation at 6 months	98 (57.6)	45 (58.4)	53 (57)	0.85
MALE 6 months	128 (75.3)	53 (68.8)	75 (80.6)	0.07
MACE 6 months	62 (36.5)	42 (67.7)	20 (21.5)	0.001
6-month mortality	53 (31.2)	39 (50.6)	14 (15.1)	0.001
Composite endpoint (Death + Major Amputation at 6 Months)	98 (57.6)	58 (75.3)	40 (43)	0.001

Quantitative variables are expressed as median (interquartile range), and categorical variables as number (percentage). MACE: major adverse cardiovascular events; MALE: major adverse limb events.

**Table 3:** Factors associated with all-cause mortality and/or major amputation at 6 months. Univariate and multivariate analysis.

	Univariate Analysis		Multivariate Analysis	
	HR (CI 95%)	*p*	HR (CI 95%)	*p*
Age > 72	1.54 (0.80–2.97)	0.195	1.59 (0.79–3.22)	0.194
Men	1.66 (1.04–2.66)	0.033	1.20 (0.57–2.51)	0.632
Rural	1.15 (0.74–1.79)	0.530		
Hypertension	1.38 (0.73–2.60)	0.324		
Diabetes mellitus	0.82 (0.50–1.34)	0.421		
Dyslipidemia	0.71 (0.46–1.11)	0.130		
Active smoker	0.72 (0.42–1.13)	0.156		
BMI > 30	1.13 (0.67–1.90)	0.660		
Alcoholism	1.03 (0.63–1.68)	0.913		
Ischemic heart disease	0.78 (0.46–1.31)	0.354		
Stroke	0.96 (0.58–1.59)	0.873		
CKD	1.05 (0.66–1.68)	0.840	1.15 (0.57–2.31)	0.690
HF	1.20 (0.76–1.91)	0.424		
AF	1.28 (0.79–2.07)	0.308		
Autoimmune disease	0.70 (0.26–1.92)	0.489		
COPD	1.06 (0.56–2.00)	0.860		
Depression	2.11 (1.20–3.71)	0.009	3.23 (1.38–7.57)	0.007
Cancer history	1.38 (0.81–2.36)	0.236		
Sleep apnea	0.81 (0.33–2.00)	0.646		
Sarcopenia	2.05 (1.31–3.20)	0.002	1.95 (1.01–3.79)	0.048
Suprapopliteal and infrapopliteal lesion	1.35 (0.87–2.11)	0.180		
Fontaine stage IV	1.14 (0.73–1.78)	0.564		
Infection	1.36 (0.87–2.14)	0.176		

AF: atrial fibrillation; BMI: body mass index; CI 95%: chronic obstructive pulmonary disease; CKD chronic kidney disease; COPD: chronic obstructive pulmonary disease; HF: heart failure; HR: hazard ratio.

## Data Availability

De-identified data are available on request preceded by a signed data access agreement form.

## Abbreviations

BIA	Bioelectrical impedance analysis
BMI	Body Mass Index
COPD	Chronic Obstructive Pulmonary Disease
CT	Computed Tomography
CTLI	Critical Limb Ischemia
DXA	Dual-energy X-ray Absorptiometry
GLASS	(Global limb Anatomic Stating System)
MACEs	Major Adverse Cardiovascular Events
MALEs	Major Adverse Limb Events
MNA-SF	Mini Nutritional Assessment–Short Form
MRI	Magnetic Resonance Imaging
PAD	Peripheral Arterial Disease
SARC-F	Strength, Assistance in walking, Rise from a chair, Climb stairs, Falls
SET	Supervised Exercise Therapy
WIFI	(Wound, Ischemia, and Foot Infection)

## References

[1] Mazzolai L., Teixido-Tura G., Lanzi S., Boc V., Bossone E., Brodmann M., Bura-Rivière A., De Backer J., Deglise S., Della Corte A. (2024). 2024 ESC Guidelines for the Management of Peripheral Arterial and Aortic Diseases. Eur. Heart J..

[2] Armstrong E.J., Armstrong D.G. (2021). Critical Limb Ischemia. Vasc. Med..

[3] Mills J.L., Conte M.S., Armstrong D.G., Pomposelli F.B., Schanzer A., Sidawy A.N., Andros G. (2014). The Society for Vascular Surgery Lower Extremity Threatened Limb Classification System: Risk Stratification Based on Wound, Ischemia, and Foot Infection (WIfI). J. Vasc. Surg..

[4] Conte M.S., Bradbury A.W., Kolh P., White J.V., Dick F., Fitridge R., Mills J.L., Ricco J.B., Suresh K.R., Murad M.H. (2019). Global Vascular Guidelines on the Management of Chronic Limb-Threatening Ischemia. J. Vasc. Surg..

[5] Taniguchi R., Deguchi J., Hashimoto T., Sato O. (2019). Sarcopenia as a Possible Negative Predictor of Limb Salvage in Patients with Chronic Limb-Threatening Ischemia. Ann. Vasc. Dis..

[6] Cruz-Jentoft A.J., Bahat G., Bauer J., Boirie Y., Bruyère O., Cederholm T., Cooper C., Landi F., Rolland Y., Aihie A. (2019). Sarcopenia: Revised European Consensus on Definition and Diagnosis. Age Ageing.

[7] Ferreira J.M.M., Cunha P., Carneiro A., Vila I., Cunha C., Silva C., Longatto-Filho A., Mesquita A., Cotter J., Mansilha A. (2021). Sarcopenia as a Prognostic Factor in Peripheral Arterial Disease: Descriptive Review. Ann. Vasc. Surg..

[8] Engin M., As A.K., Aydın U., Ata Y. (2024). Mortality and Morbidity Risk Factors in Patients with Critical Limb Ischemia. Vascular.

[9] Sayer A.A., Cruz-Jentoft A. (2022). Sarcopenia Definition, Diagnosis and Treatment: Consensus Is Growing. Age Ageing.

[10] Ravindhran B., Igwe C., Nazir S., Harwood A.E., Lathan R., Carradice D., Smith G.E., Chetter I.C., Pymer S. (2025). The Association Between Completion of Supervised Exercise Therapy and Long-Term Outcomes in Patients with Intermittent Claudication, Concomitant Sarcopenia, and Cardiometabolic Multimorbidity. Ann. Vasc. Surg..

[11] Pizzimenti M., Meyer A., Charles A.L., Giannini M., Chakfé N., Lejay A., Geny B. (2020). Sarcopenia and Peripheral Arterial Disease: A Systematic Review. J. Cachexia Sarcopenia Muscle.

[12] Cao Z., Zhao B., Jiang T., Zhang T., Yu X., Li Y., Wu W. (2023). Association of Sarcopenia with Mortality in Patients with Chronic Limb-Threatening Ischemia Undergoing Endovascular Revascularization. J. Surg. Res..

[13] Selçuk N., Albeyoğlu Ş., Bastopcu M., Selçuk İ., Barutca H., Şahan H. (2023). Sarcopenia Is a Risk Factor for Major Adverse Cardiac Events after Surgical Revascularization for Critical Limb Ischemia. Vascular.

[14] Sivaharan A., Boylan L., Witham M.D., Nandhra S. (2021). Sarcopenia in Patients Undergoing Lower Limb Bypass Surgery Is Associated with Higher Mortality and Major Amputation Rates. Ann. Vasc. Surg..

[15] Bradley N.A., Walter A., Roxburgh C.S.D., McMillan D.C., Guthrie G.J.K. (2024). The Relationship between Clinical Frailty Score, CT-Derived Body Composition, Systemic Inflammation, and Survival in Patients with Chronic Limb-Threatening Ischemia. Ann. Vasc. Surg..

[16] Barazzoni R., Jensen G.L., Correia M.I.T.D., Gonzalez M.C., Higashiguchi T., Shi H.P., Bischoff S.C., Boirie Y., Carrasco F., Cruz-Jentoft A. (2022). Guidance for Assessment of the Muscle Mass Phenotypic Criterion for the Global Leadership Initiative on Malnutrition (GLIM) Diagnosis of Malnutrition. Clin. Nutr..

[17] Sánchez-Rodríguez D., De Meester D., Minon L., Claessens M., Gümüs N., Lieten S., Benoit F., Surquin M., Marco E. (2023). Association between Malnutrition Assessed by the Global Leadership Initiative on Malnutrition Criteria and Mortality in Older People: A Scoping Review. Int. J. Environ. Res. Public Health.

[18] Miyahara S., Maeda K., Yasuda A., Satake S., Arai H. (2024). The Potential of Body Mass Index-Adjusted Calf Circumference as a Proxy for Low Muscle Mass in the Global Leadership Initiative on Malnutrition Criteria. Clin. Nutr..

[19] Ferreira J., Carneiro A., Vila I., Silva C., Cunha C., Longatto-Filho A., Mesquita A., Cotter J., Mansilha A., Correia-Neves M. (2023). Inflammation and Loss of Skeletal Muscle Mass in Chronic Limb Threatening Ischemia. Ann. Vasc. Surg..

[20] Lebreton O., Fels A., Compagnon A., Lazareth I., Ghaffari P., Chatellier G., Emmerich J., Michon-Pasturel U., Priollet P., Yannoutsos A. (2023). Amputation-Free Survival in the Long-Term Follow-up and Gender-Related Characteristics in Patients Revascularized for Critical Limb Ischemia. JMV-J. Med. Vasc..

[21] Zielke T., Korepta L., Wesolowski M., D’Andrea M., Aulivola B. (2024). The Association of Comorbid Depression with Mortality and Amputation Risk in Patients with Chronic Limb-Threatening Ischemia. J. Vasc. Surg..

[22] Harris K.M., Mena-Hurtado C., Burg M.M., Vriens P.W., Heyligers J., Smolderen K.G. (2023). Association of Depression and Anxiety Disorders with Outcomes after Revascularization in Chronic Limb-Threatening Ischemia Hospitalizations Nationwide. J. Vasc. Surg..

[23] Zahner G.J., Cortez A., Duralde E., Ramirez J.L., Wang S., Hiramoto J., Cohen B.E., Wolkowitz O.M., Arya S., Hills N.K. (2020). Association of Comorbid Depression with Inpatient Outcomes in Critical Limb Ischemia. Vasc. Med..

[24] Casajuana Urgell E., Calsina Juscafresa L., Nieto Fernandez L., Romero Montaña L., Llort Pont C., Clarà Velasco A. (2022). Critical Limb Ischemia in Nonagenarians: A Challenge of Our Times. World J. Surg..

[25] Froud J.L.J., Landin M., Wafi A., White S., Bearne L., Patel A., Modarai B. (2025). Rate and Predictors of Disease Progression in Patients with Conservatively Managed Intermittent Claudication: A Systematic Review. Ann. Vasc. Surg..

[26] Morisaki K., Furuyama T., Matsubara Y., Inoue K., Kurose S., Yoshino S., Nakayama K., Yamashita S., Yoshiya K., Mori M. (2020). Thigh Sarcopenia and Hypoalbuminemia Predict Impaired Overall Survival after Infrainguinal Revascularization in Patients with Critical Limb Ischemia. Vascular.

[27] Gornik H.L., Aronow H.D., Goodney P.P., Arya S., Brewster L.P., Byrd L., Chandra V., Drachman D.E., Eaves J.M., Ehrman J.K. (2024). 2024 ACC/AHA/AACVPR/APMA/ABC/SCAI/SVM/SVN/SVS/SIR/VESS Guideline for the Management of Lower Extremity Peripheral Artery Disease: A Report of the American College of Cardiology/American Heart Association Joint Committee on Clinical Practice Guidelines. Circulation.

[28] Jansen S.C.P., Hoorweg B.B.N., Hoeks S.E., van den Houten M.M.L., Scheltinga M.R.M., Teijink J.A.W., Rouwet E.V. (2019). A Systematic Review and Meta-Analysis of the Effects of Supervised Exercise Therapy on Modifiable Cardiovascular Risk Factors in Intermittent Claudication. J. Vasc. Surg..

[29] Noetel M., Sanders T., Gallardo-Gómez D., Taylor P., Del Pozo Cruz B., Van Den Hoek D., Smith J.J., Mahoney J., Spathis J., Moresi M. (2024). Effect of Exercise for Depression: Systematic Review and Network Meta-Analysis of Randomised Controlled Trials. BMJ.

[30] Pearce M., Garcia L., Abbas A., Strain T., Schuch F.B., Golubic R., Kelly P., Khan S., Utukuri M., Laird Y. (2022). Association between Physical Activity and Risk of Depression: A Systematic Review and Meta-Analysis. JAMA Psychiatry.

[31] Söderlund M., Huhtamo H., Protto S., Hernesniemi J.A., Vakhitov D., Oksala N., Khan N. (2024). Magnetic Resonance Imaging—Derived Psoas Muscle Area and Survival in Patients Treated Invasively for Peripheral Arterial Disease. Scand. J. Surg..

